# Opportunities and Challenges for Clinical Practice in Detecting Depression Using EEG and Machine Learning

**DOI:** 10.3390/s25020409

**Published:** 2025-01-12

**Authors:** Damir Mulc, Jaksa Vukojevic, Eda Kalafatic, Mario Cifrek, Domagoj Vidovic, Alan Jovic

**Affiliations:** 1 University Psychiatric Hospital Vrapče, Bolnička Cesta 32, 10000 Zagreb, Croatia; damir.mulc@bolnica-vrapce.hr (D.M.); jaksa.vukojevic@bolnica-vrapce.hr (J.V.); domagoj.vidovic@bolnica-vrapce.hr (D.V.); 2 University of Zagreb Faculty of Electrical Engineering and Computing, Unska 3, 10000 Zagreb, Croatia; eda.kalafatic@fer.unizg.hr (E.K.); mario.cifrek@fer.unizg.hr (M.C.)

**Keywords:** depression detection, major depressive disorder, electroencephalography, machine learning

## Abstract

Major depressive disorder (MDD) is associated with substantial morbidity and mortality, yet its diagnosis and treatment rates remain low due to its diverse and often overlapping clinical manifestations. In this context, electroencephalography (EEG) has gained attention as a potential objective tool for diagnosing depression. This study aimed to evaluate the effectiveness of EEG in identifying MDD by analyzing 140 EEG recordings from patients diagnosed with depression and healthy volunteers. Using various machine learning (ML) classification models, we achieved up to 80% accuracy in distinguishing individuals with MDD from healthy controls. Despite its promise, this approach has limitations. The variability in the clinical and biological presentations of depression, as well as patient-specific confounding factors, must be carefully considered when integrating ML technologies into clinical practice. Nevertheless, our findings suggest that an EEG-based ML model holds potential as a diagnostic aid for MDD, paving the way for further refinement and clinical application.

## 1. Introduction

The World Health Organization (WHO) has recognized major depressive disorder (MDD) as one of the most common causes of disability worldwide [1]. It is characterized by a diverse array of symptoms [2], which together pose a major challenge for accurate diagnosis and effective management [3]. This heterogeneity of clinical presentations is also reflected in patients’ varied and sometimes unpredictable responses to standard pharmacological and psychotherapeutic interventions, further complicating treatment planning and the predictability of outcomes [4]. Still, the diagnosis of MDD relies primarily on diagnostic criteria and then on the clinician’s subjective assessment of the severity of symptoms using interviews and standardized clinical scales. While diagnostic systems aim to provide clarity and consistency in diagnosing MDD, they also face notable limitations and challenges [5], like the potential for overlap, false positives, or missed diagnoses, particularly when presented symptoms mimic those of other psychiatric or neurological conditions. For instance, symptoms of MDD are seen in bipolar disorder [6], and resemble some of those often seen in PTSD [7], personality disorders [8], or even early dementia [9]. This lack of differentiation in the nuanced symptomatology has been criticized because it contributes to diagnostic inaccuracies [10]. Critics have also questioned the empirical basis for the thresholds used to diagnose MDD, suggesting that they sometimes pathologize transient or typical emotional states as major depressive episodes [11]. Both the DSM-5 and the ICD prioritize reliability (and thus consistent application by different clinicians) over validity (or the ability of diagnoses to accurately reflect underlying conditions), so this emphasis on categorization can lead to diagnoses that are not necessarily tailored to the individual patient [5]. Furthermore, diagnostic criteria do not adequately reflect the impact of individual symptoms on overall clinical severity. For example, suicidal ideation and anhedonia are more strongly associated with the seriousness of depression, while somatic symptoms often overlap with those from a physical illness [12]. Also, the above-mentioned heterogeneity of MDD—characterized by overlapping symptoms, varying severity, different onset patterns, and fluctuating disease courses—also leads to a broad spectrum of clinical subtypes [13,14,15]. Finally, the diagnostic process is complicated by the variability in the presentation of MDD across different populations [16], posing further challenges to the objectivity and consistency of diagnostic systems.

With this in mind, the persistent need for more objective diagnostic tools has led researchers to investigate electroencephalography (EEG), a widely used non-invasive neuroimaging technique, as a means of identifying biomarkers for the diagnosis and prediction of treatment outcome in MDD [17,18,19]. A prominent contemporary approach involves employing machine learning (ML) classification algorithms to predict diagnoses based on selected features [20]. To date, numerous studies have explored various signal analysis methods and data processing techniques and have gained valuable insights during the process [21]. Among these, biomarkers derived from the alpha band have consistently demonstrated efficacy [22,23,24], with additional evidence supporting the roles of the gamma [25] and theta bands [26]. Moreover, interhemispheric frontal alpha asymmetry has shown promise, particularly in predicting treatment outcomes [27,28,29]. However, the success and reliability of this approach are highly dependent on effective feature selection [30] and access to robust, high-quality training data, which is particularly crucial for complex conditions such as depression [31,32]. In summary, although no final consensus has been reached, the recurring similarities between the biomarkers identified suggest promising directions for further research and refinements in this field.

The described heterogeneity of previous studies with similar designs, the difficulties in assessing their transparency and the quality of the results (due to the variations in datasets, feature selection, ML algorithms used, signal processing techniques, metrics used to show the results, etc.), the lack of a standardized approach, and the risk of their purely academic utility pose significant challenges. The aim of this study was to create a method for detecting depression using EEG recordings of individuals and patients in the clinical setting using standardized equipment and to recognize the difficulties and the possible potential of this approach for daily practice. We discuss the use of the method and its impact on diagnosis and outcomes.

## 2. Materials and Methods

Diagnosing MDD based on EEG represents a potential diagnostic tool, so we aimed to test several ML classification models on EEG recordings from patients diagnosed with MDD and healthy volunteers. The research was conducted in the following steps:

1. Dataset acquisition;
2. Preprocessing;
3. Feature extraction;
4. Classification.

### 2.1. Dataset Acquisition

The dataset was recorded at the University Psychiatric Hospital Vrapče, Zagreb, Croatia. The dataset consists of a total of 140 EEG recordings from adult (>18-year-old) patients (*n* = 70), recorded as part of the standardized diagnostic procedure, and healthy volunteers (*n* = 70). Patients were diagnosed with moderate-to-severe MDD, with diagnosis and severity determined by senior psychiatrists according to ICD-10 criteria. Recordings from patients diagnosed with any other comorbid psychiatric condition besides personality disorders were excluded from the study, as well as patients with neurological and “somatic” diagnoses (besides hypertension and hyperlipidemia). In the same setting and with the same protocol as for patients, we also recorded EEGs from 70 volunteers, from which we obtained written informed consent, and with whom a psychiatric interview was conducted to exclude psychiatric and neurological disorders as well as psychopharmacotherapy use. This study was conducted in accordance with the guidelines of the Declaration of Helsinki and approved by the Ethics Committee of the University Psychiatric Hospital Vrapče.

The subjects were age- and sex-matched to the best extent (Table 1). Still, there were more depressed female subjects than male subjects for the exact number of subjects per sex and mean age values for all groups.

**Figure 1:**
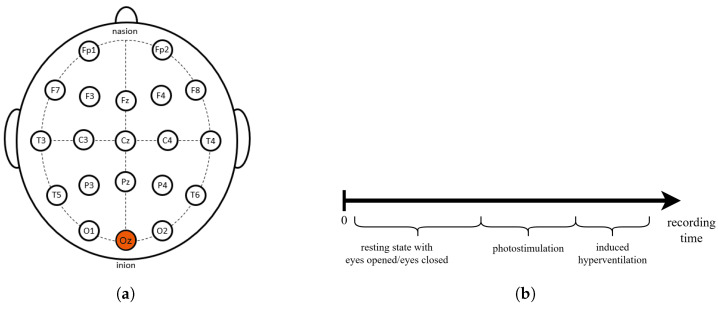
(**a**) The 10–20 system with 19 channels. (**b**) EEG recording protocol.

EEGs were recorded using a 19-channel EEG amplifier, using the standard 10–20 electrode array, and Oz as the reference electrode, as shown in Figure 1a. The sampling frequency was 200 Hz. Subjects were in a comfortable lying-down position, and the room was kept quiet and peaceful. The recording lasted 30 min in total for each subject, during which they followed the instructions of the technician who carried out the recording. During the recording, the technician noted all events (opened or closed eyes, photostimulation frequency, etc.). Other important events such as subjects’ movement, swallowing, or blinking were also marked by the technician if they interfered with the EEG recording. The recording protocol consisted of a resting-state EEG with interchanging periods of opened and closed eyes, followed by photostimulation with 5 different flash frequencies (4 Hz, 8 Hz, 16 Hz, 24 Hz, and 30 Hz), and induced hyperventilation (Figure 1b).

### 2.2. Preprocessing

**Figure 2:**
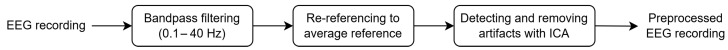
EEG preprocessing protocol.

Raw EEG data are usually contaminated with artifacts and may not accurately reflect the underlying brain activity, so preprocessing of the data is required. The preprocessing was performed using Matlab R2021b version EEGLAB toolbox. To remove the noise, the signal was first filtered, then re-referenced to the average reference, and finally analyzed with independent component analysis (ICA) to remove artifacts (Figure 2).

EEG signals were filtered with a bandpass FIR filter with cutoff frequencies of 0.1 and 40 Hz. The lower-frequency range was chosen to eliminate the slow drifts in the signal [33], while the upper-frequency range was chosen to preserve the frequency components of interest while removing the 50 Hz conduction noise. The signals were then re-referenced to the average reference [34]. ICA was performed to decompose and analyze the signal. Each component was labeled using ICLabel, an algorithm that classifies each component by its source. Possible labels were brain, eye, muscle, and line noise, which provided an estimation of the type of components such as brain, eye, muscle, heart, line noise, signal noise, and other [35]. Components classified as artifacts were then manually inspected, and their subsets were subsequently removed to find a minimal subset that removed the noticeable artifacts while preserving the components that make up the valuable part of the signal.

### 2.3. Feature Extraction

For further analysis of using EEG signals as a potential diagnostic tool for depression, only resting-state recording was used instead of the whole 30 min recording, as this part contains the lowest number of artifacts and is the most commonly used protocol in work on affective disorders [36]. From each subject, exactly five interchanging periods of opened and closed eyes were extracted and used in the next step—feature extraction. Feature extraction was performed using Matlab R2021b version.

The EEG signal was decomposed using wavelet transformation into five primary characteristic brain waves: alpha, beta, gamma, theta, and delta. Six features were selected and extracted from each of the 19 available EEG channels, giving a total of 570 features per subject. We selected both linear and nonlinear features [37]: absolute and relative band power [17,38], spectral centroids [39], relative wavelet energy, wavelet entropy [40,41], and Katz Fractal Dimension [42]. The framework for feature extraction is more thoroughly explained in our previous work [43].

### 2.4. Classification

Training and testing of machine learning models were conducted in Python using the pandas and scikit-learn libraries. The dataset was randomly split into a training set (100 subjects) and a test set (40 subjects) with stratification based on diagnosis, this way ensuring that both sets contained the same ratio of depressed and healthy subjects. There was no overlap between the training and test sets, and the data were split subject-wise.

Six ML models were trained and tested: decision tree (DT), support vector machine (SVM), random forest (RF), K-nearest neighbor (KNN), eXtreme Gradient Boosting (XGBoost), and Naïve Bayes (NB). For each model, tuning of the hyperparameters was performed using grid search with 10-fold cross-validation on the training set. The results of the tuning (best hyperparameters) for each model are shown in Table 2.

Dimensionality reduction in machine learning helps eliminate irrelevant data, noise, and redundant features, improving accuracy and reducing training time [44]. Since one of the key points in building a potential diagnostic tool is choosing the right features as potential biomarkers for MDD, we tested our models on both the full dataset (570 features) and the reduced dataset (100 features). Mutual information was used as a criterion for selecting features to reduce the dimension of the dataset.

## 3. Results

**Figure 3:**
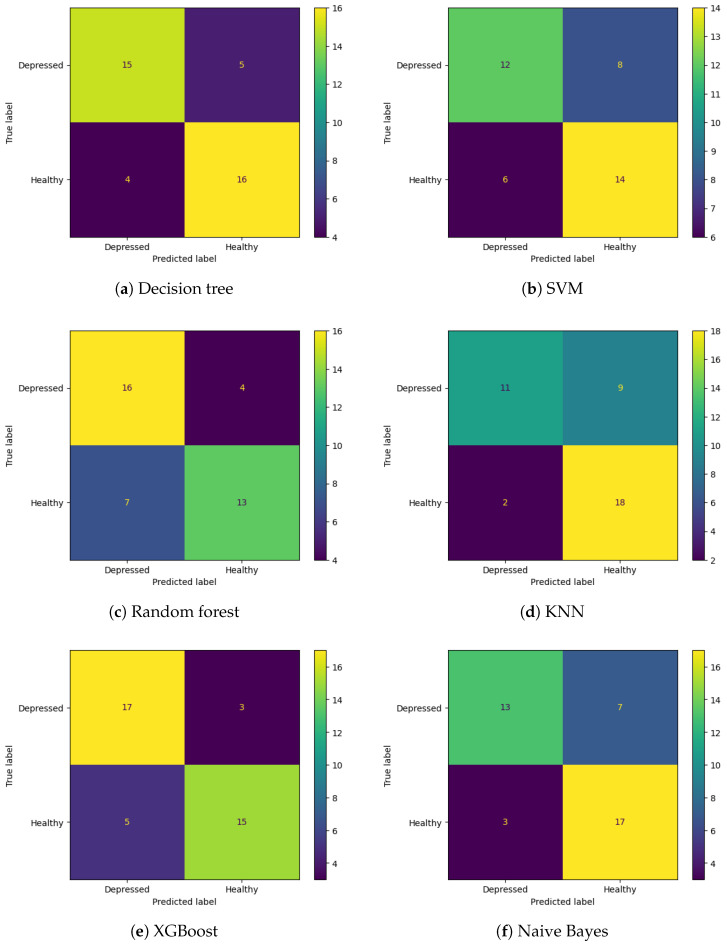
Confusion matrices for tested ML models.

We evaluated all the applied machine learning algorithms using two metrics, accuracy and F1-score [45], and compared results when using a full dataset (all features) and a reduced dataset (100 features). The test set confusion matrices for each model are shown in Figure 3.

From the confusion matrices, it can be observed that SVM, KNN, and Naive Bayes have a higher number of false positives, meaning that healthy subjects were misclassified as depressed subjects, whereas random forest has more false negatives (depressed subjects classified as healthy). Decision tree and XGBoost had a similar number of false positives and false negatives.

The best classification results were achieved by XGBoost with an accuracy of 80% and an F1-score of 0.81, followed by a decision tree with an accuracy of 78% and an F1-score of 0.77 on the full features test dataset. In comparison, the reduced features dataset had slightly worse results, where the decision tree had an accuracy of 78% and an F1-score of 0.79, and XGBoost had an accuracy of 75% and an F1-score of 0.77. The classification results for all of the models and both datasets are shown in Table 3.

## 4. Discussion

For some time now, researchers have been trying to separate healthy from depressed individuals using EEG, thus providing a potential diagnostic biomarker [46,47]. Although this approach shows promise as a clinical decision support system [48], it also brings some potential pitfalls. Most of them are related to the nature of psychiatric disorders, characterized by heterogeneity and complex etiology [49]. In any case, in this type of task, we use a clinical-based approach to categorically define groups and then use this to build an ML classification model.

The use of data from the hospital database provided us with a reliable sample size, but also with confounding factors whose importance needs to be considered in future research or possible clinical implementation. Among the most important is the presence of comorbid psychiatric disorders, such as anxiety disorders [50,51] or personality disorders [52], together with pharmacological treatment [53]. Other factors, such as situational anxiety [54], hormonal (menstrual) status [55], fatigue and sleepiness [56,57], or even time of day [58] during recording, could be effectively overcome by standardizing the protocols in both clinical practice and study designs. In future work, exploration of which recording condition (resting state with eyes open or eyes closed; photostimulation; hyperventilation; or other possible task paradigms, such as eliciting the P300) should be carried out to determine the best recording protocol for depression detection. This was already performed for the two conditions of eyes open and eyes closed, suggesting that there is more information about the altered EEG in depression in the eyes open resting state [59].

Moreover, due to the nature of EEG signals which are non-stationary and stochastic, meaning the signals contain some nonlinear characteristics [60], our classification was based on both linear and nonlinear features. In this study, absolute and relative band power, spectral centroid, relative wavelet energy, and wavelet entropy were used as linear features because they are good predictors [17,38,43]. On the other hand, the nonlinear feature used, the Katz Fractal Dimension, is possibly more appropriate for the “underlying” nonlinear brain activity [61,62]. Also, it is important to note that gender differences have been found with regard to the EEG signal in depression [63,64], in addition to those related to the subject’s age [65]. It is therefore important that there is a balanced ratio between the age and gender of the healthy volunteers and the depressed test subjects in both the training and the test group.

We provide a quantitative comparison with other studies in Table 4. Compared to the other studies, where the sample size is usually between 15 and 60 subjects [24,66,67,68], our dataset consists of 140 subjects. A study from China had a larger dataset of 200 MDD and 200 healthy subjects and their results were similar to ours with the best accuracy obtained after sequential backward feature selection of 84%, whereas our best accuracy was 80% [69]. Both our study and the study by [69] show weaker results in terms of accuracy compared to studies with a smaller number of subjects. This is a common and consistently reoccurring problem in psychiatry [70]. We also note that the studies are mostly incomparable since they were performed on different datasets, most of them private. The compared studies were chosen based on the used ML models, as comparison with deep learning models would not be appropriate due to the significantly different methodologies.

We should also be cautious when looking at clinical outcomes using specific metrics, as there could be differences in the interpretation of results when different metrics are used. In a clinical context, it is crucial to prioritize an accurate identification of patients suffering from MDD, which means that a high hit rate (i.e., “capturing” the majority of depressed individuals) would be of greatest benefit. However, sacrificing precision in this process could lead to more false positives, which could subsequently affect the course of treatment and prognosis of inaccurately diagnosed patients. As previously mentioned, many types of adjustment disorders (often a part of permanent personality disorder) [64], or those associated with anxiety [71], neurodegeneration [72], bipolar [73], or even disorders from the schizophrenia spectrum [74], may exhibit symptoms similar to those in MDD. For this reason, we believe that it is best to use a combined score to demonstrate the reliability of these methods for classifying MDD. An overview of our results compared to primary care [75] suggests that this approach is a promising and potentially useful diagnostic aid. However, for a step in the right direction, confounding factors should not be overlooked. In addition, this search for biomarkers is deeply embedded in basic research on depression, as multidisciplinary approaches have gained increasing importance [76,77,78]. Therefore, a symptom-based approach to the recognition of MDD should adapt to understanding the disease and rely on the fact that there are different neurobiological (underlying) profiles of the disease [79], accordingly meaning that the concept of a single diagnosis should be broken down into various corresponding clinical categories, respecting the differences and limitations of various psychological scales [80]. Likewise, new ML techniques, such as deep learning classification algorithms, could lead to higher levels of accuracy [81,82,83,84], but with a loss of interpretability [85].

## 5. Conclusions

The results of our work were promising and we generally believe that the use of machine learning in the analysis of EEG signals would lead to the development of tools that can diagnose MDD adequately and thus could have a place in future daily clinical practice. Despite this hope, it is necessary to recognize and overcome many factors that may blur the diagnosis itself and provide us with inadequate conclusions. Future study designs should perhaps be guided by using more detailed medical data to correlate signs or symptoms with different types of MDD or their severity and link the process to our understanding of the illness.

## Figures and Tables

**Table 1:** Age and sex distribution of subjects.

Diagnosis	Female	Female Age (Mean ± Std)	Male	Male Age (Mean ± Std)	Total
Healthy	32	35.88 ± 9.96	38	36.16 ± 11.35	70
MDD	37	36.86 ± 10.22	33	45.24 ± 12.10	70

**Table 2:** Tuned hyperparameters for different machine learning models.

Model	Tuned Hyperparameters
Decision tree	Criterion: gini; maximum depth: 2; minimum samples per leaf: 1; minimum samples per split: 2; splitter: random
SVM	Kernel: rbf; regularization parameter (*C*): 1.0; gamma: scale
Random forest	Number of estimators: 50; criterion: gini; minimum samples per split: 10; minimum samples per leaf: 2
KNN	Number of neighbors: 5; leaf size: 30; weights: uniform
XGBoost	Learning rate: 0.5; number of estimators: 50; maximum depth: 5; gamma: 3

**Table 3:** Classification results on the full dataset (570 features) and reduced dataset (100 features).

Model	All Features (570)		Selected Features (100)	
	Accuracy	F1-Score	Accuracy	F1-Score
Decision tree	0.78	0.77	0.78	0.79
SVM	0.65	0.63	0.73	0.72
Random forest	0.73	0.74	0.73	0.74
KNN	0.73	0.67	0.60	0.50
XGBoost	**0.80**	**0.81**	0.75	0.77
Naive Bayes	0.75	0.72	0.62	0.62

**Table 4:** Comparison of classification results with other recent studies.

Research	Dataset	Features	ML Methods	Accuracy
Mahato, Paul (2020) [24]	30 MDD, 30 H	wavelet power, theta asymmetry (27 features total)	LR, SVM, NB, DT	SVM, 88.33%
Zhu et al. (2020) [67]	17 MDD, 17 H	max PSD, sumpower, activity, complexity, mobility, variance, mean square, different entropies, correlation dimension, C0-complexity, Lempel-Ziv complexity (304 features total)	BayesNet, LR, RF, NB, SVM, KNN, CBEM	CBEM, 92.65%
Wu et al. (2021) [69]	200 MDD, 200 H	band power, coherence, Higuchi Fractal Dimension, Katz Fractal Dimension (1859 features total, SBS wrapper feature selection)	KNN, LDA, SVM, CK-SVM	CK-SVM, 84.16%
Avots et al. (2022) [68]	10 MDD, 10 H	relative band power, alpha power variability, spectral asymmetry index, Higuchi Fractal Dimension (162 features total, ReliefF feature selection)	SVM, LDA, NB, KNN, DT, ensemble	KNN, 95.00%
Our work	70MDD, 70H	absolute and relative band power, spectral centroid, RWE, WE, Katz Fractal Dimension (570 features total, mutual information feature selection)	DT, SVM, RF, KNN, XGBoost	XGBoost, 80.00%

## Data Availability

Data available on request.

## Abbreviations

The following abbreviations are used in this manuscript:

MDD	major depressive disorder
EEG	electroencephalography
ML	machine learning
ICD-10	International Classification of Diseases, Tenth Revision
DSM-5	*The Diagnostic and Statistical Manual of Mental Disorders, Fifth Edition*
ICA	independent component analysis
FIR	finite impulse response
DT	decision tree
SVM	support vector machine
KNN	K-nearest neighbors
XGBoost	eXtreme Gradient Boosting
NB	Naïve Bayes
PSD	power spectral density
RWE	relative wavelet energy
WE	wavelet entropy

## References

[1] James S.L., Abate D., Abate K.H., Abay S.M., Abbafati C., Abbasi N., Abbastabar H., Abd-Allah F., Abdela J., Abdelalim A. (2018). Global, regional, and national incidence, prevalence, and years lived with disability for 354 diseases and injuries for 195 countries and territories, 1990–2017: A systematic analysis for the Global Burden of Disease Study 2017. Lancet.

[2] Zimmerman M., Ellison W., Young D., Chelminski I., Dalrymple K. (2015). How many different ways do patients meet the diagnostic criteria for major depressive disorder?. Compr. Psychiatry.

[3] Nelson G.H., O’Hara M.W., Watson D. (2018). National norms for the expanded version of the inventory of depression and anxiety symptoms (IDAS-II). J. Clin. Psychol..

[4] Cuijpers P., Noma H., Karyotaki E., Vinkers C.H., Cipriani A., Furukawa T.A. (2020). A network meta-analysis of the effects of psychotherapies, pharmacotherapies and their combination in the treatment of adult depression. World Psychiatry.

[5] Kendler K.S. (2016). The phenomenology of major depression and the representativeness and nature of DSM criteria. Am. J. Psychiatry.

[6] Kessing L.V., González-Pinto A., Fagiolini A., Bechdolf A., Reif A., Yildiz A., Etain B., Henry C., Severus E., Reininghaus E.Z. (2021). DSM-5 and ICD-11 criteria for bipolar disorder: Implications for the prevalence of bipolar disorder and validity of the diagnosis—A narrative review from the ECNP bipolar disorders network. Eur. Neuropsychopharmacol..

[7] Barbano A.C., van der Mei W.F., deRoon Cassini T.A., Grauer E., Lowe S.R., Matsuoka Y.J., O’Donnell M., Olff M., Qi W., Ratanatharathorn A. (2019). Differentiating PTSD from anxiety and depression: Lessons from the ICD-11 PTSD diagnostic criteria. Depress. Anxiety.

[8] Beatson J.A., Rao S. (2013). Depression and borderline personality disorder. Med. J. Aust..

[9] Rubin R. (2018). Exploring the relationship between depression and dementia. JAMA.

[10] Cerbo A. (2021). Convergences and divergences in the ICD-11 vs. DSM-5 classification of mood disorders. Turk. Psikiyatr. Derg. Turk. J. Psychiatry.

[11] Horwitz A.V., Wakefield J.C. (2007). The Loss of Sadness: How Psychiatry Transformed Normal Sorrow into Depressive Disorder.

[12] Zimmerman M., Balling C., Chelminski I., Dalrymple K. (2018). Understanding the severity of depression: Which symptoms of depression are the best indicators of depression severity?. Compr. Psychiatry.

[13] Rush A.J. (2007). The varied clinical presentations of major depressive disorder. J. Clin. Psychiatry.

[14] Fried E.I. (2017). The 52 symptoms of major depression: Lack of content overlap among seven common depression scales. J. Affect. Disord..

[15] Musil R., Seemüller F., Meyer S., Spellmann I., Adli M., Bauer M., Kronmüller K.T., Brieger P., Laux G., Bender W. (2018). Subtypes of depression and their overlap in a naturalistic inpatient sample of major depressive disorder. Int. J. Methods Psychiatr. Res..

[16] Kessler R.C., Bromet E.J. (2013). The epidemiology of depression across cultures. Annu. Rev. Public Health.

[17] Hosseinifard B., Moradi M.H., Rostami R. (2013). Classifying depression patients and normal subjects using machine learning techniques and nonlinear features from EEG signal. Comput. Methods Programs Biomed..

[18] Mumtaz W., Xia L., Ali S.S.A., Yasin M.A.M., Hussain M., Malik A.S. (2017). Electroencephalogram (EEG)-based computer-aided technique to diagnose major depressive disorder (MDD). Biomed. Signal Process. Control.

[19] Cai H., Han J., Chen Y., Sha X., Wang Z., Hu B., Yang J., Feng L., Ding Z., Chen Y. (2018). A pervasive approach to EEG-based depression detection. Complexity.

[20] Aleem S., Huda N.u., Amin R., Khalid S., Alshamrani S.S., Alshehri A. (2022). Machine learning algorithms for depression: Diagnosis, insights, and research directions. Electronics.

[21] de Aguiar Neto F.S., Rosa J.L.G. (2019). Depression biomarkers using non-invasive EEG: A review. Neurosci. Biobehav. Rev..

[22] Jaworska N., Blier P., Fusee W., Knott V. (2012). Alpha power, alpha asymmetry and anterior cingulate cortex activity in depressed males and females. J. Psychiatr. Res..

[23] Shim M., Im C.H., Kim Y.W., Lee S.H. (2018). Altered cortical functional network in major depressive disorder: A resting-state electroencephalogram study. NeuroImage Clin..

[24] Mahato S., Paul S. (2020). Classification of depression patients and normal subjects based on electroencephalogram (EEG) signal using alpha power and theta asymmetry. J. Med. Syst..

[25] Fitzgerald P.J., Watson B.O. (2018). Gamma oscillations as a biomarker for major depression: An emerging topic. Transl. Psychiatry.

[26] Dharmadhikari A., Tandle A., Jaiswal S., Sawant V., Vahia V., Jog N. (2018). Frontal theta asymmetry as a biomarker of depression. East Asian Arch. Psychiatry.

[27] Kaiser A.K., Gnjezda M.T., Knasmüller S., Aichhorn W. (2018). Electroencephalogram alpha asymmetry in patients with depressive disorders: Current perspectives. Neuropsychiatr. Dis. Treat..

[28] Reznik S.J., Allen J.J. (2018). Frontal asymmetry as a mediator and moderator of emotion: An updated review. Psychophysiology.

[29] Van Der Vinne N., Vollebregt M.A., Van Putten M.J., Arns M. (2017). Frontal alpha asymmetry as a diagnostic marker in depression: Fact or fiction? A meta-analysis. NeuroImage Clin..

[30] Cukic M., Pokrajac D., Stokic M., Radivojevic V., Ljubisavljevic M. (2018). EEG machine learning with Higuchi fractal dimension and Sample Entropy as features for successful detection of depression. arXiv.

[31] Wilkinson J., Arnold K.F., Murray E.J., van Smeden M., Carr K., Sippy R., de Kamps M., Beam A., Konigorski S., Lippert C. (2020). Time to reality check the promises of machine learning-powered precision medicine. Lancet Digit. Health.

[32] Chen Z.S., Galatzer-Levy I.R., Bigio B., Nasca C., Zhang Y. (2022). Modern views of machine learning for precision psychiatry. Patterns.

[33] Sanei S., Chambers J.A. (2013). EEG Signal Processing.

[34] Mumtaz W., Malik A.S., Ali S.S.A., Yasin M.A.M. (2015). A Study to Investigate Different EEG Reference Choices in Diagnosing Major Depressive Disorder. Proceedings of the Neural Information Processing: 22nd International Conference, ICONIP 2015.

[35] Pion-Tonachini L., Kreutz-Delgado K., Makeig S. (2019). ICLabel: An automated electroencephalographic independent component classifier, dataset, and website. NeuroImage.

[36] Damborská A., Tomescu M.I., Honzírková E., Barteček R., Hořínková J., Fedorová S., Ondruš Š., Michel C.M. (2019). EEG resting-state large-scale brain network dynamics are related to depressive symptoms. Front. Psychiatry.

[37] Mahato S., Paul S. (2019). Detection of major depressive disorder using linear and non-linear features from EEG signals. Microsyst. Technol..

[38] Mohammadi M., Al-Azab F., Raahemi B., Richards G., Jaworska N., Smith D., de la Salle S., Blier P., Knott V. (2015). Data mining EEG signals in depression for their diagnostic value. BMC Med. Inform. Decis. Mak..

[39] Kulkarni N. (2018). Use of complexity based features in diagnosis of mild Alzheimer disease using EEG signals. Int. J. Inf. Technol..

[40] Bairy G.M., Niranjan U., Puthankattil S.D. (2016). Automated classification of depression EEG signals using wavelet entropies and energies. J. Mech. Med. Biol..

[41] Puthankattil S.D., Joseph P.K. (2014). Analysis of EEG signals using wavelet entropy and approximate entropy: A case study on depression patients. Int. J. Bioeng. Life Sci..

[42] Akar S.A., Kara S., Agambayev S., Bilgiç V. (2015). Nonlinear analysis of EEG in major depression with fractal dimensions. Proceedings of the 2015 37th Annual International Conference of the IEEE Engineering in Medicine and Biology Society (EMBC).

[43] Kinder I., Friganovic K., Vukojevic J., Mulc D., Slukan T., Vidovic D., Brecic P., Cifrek M. (2020). Comparison of machine learning methods in classification of affective disorders. Proceedings of the 2020 43rd International Convention on Information, Communication and Electronic Technology (MIPRO).

[44] Zebari R., Abdulazeez A., Zeebaree D., Zebari D., Saeed J. (2020). A comprehensive review of dimensionality reduction techniques for feature selection and feature extraction. J. Appl. Sci. Technol. Trends.

[45] Obi J.C. (2023). A comparative study of several classification metrics and their performances on data. World J. Adv. Eng. Technol. Sci..

[46] Bhadra S., Kumar C.J. (2022). An insight into diagnosis of depression using machine learning techniques: A systematic review. Curr. Med. Res. Opin..

[47] Pinto S.J., Parente M. (2024). Comprehensive review of depression detection techniques based on machine learning approach. Soft Comput..

[48] Kessler R.C. (2018). The potential of predictive analytics to provide clinical decision support in depression treatment planning. Curr. Opin. Psychiatry.

[49] Buch A.M., Liston C. (2021). Dissecting diagnostic heterogeneity in depression by integrating neuroimaging and genetics. Neuropsychopharmacology.

[50] Nusslock R., Shackman A.J., McMenamin B.W., Greischar L.L., Davidson R.J., Kovacs M. (2018). Comorbid anxiety moderates the relationship between depression history and prefrontal EEG asymmetry. Psychophysiology.

[51] Lin I.M., Chen T.C., Lin H.Y., Wang S.Y., Sung J.L., Yen C.W. (2021). Electroencephalogram patterns in patients comorbid with major depressive disorder and anxiety symptoms: Proposing a hypothesis based on hypercortical arousal and not frontal or parietal alpha asymmetry. J. Affect. Disord..

[52] Vukojević J., Mulc D., Kinder I., Jovičić E., Friganović K., Savić A., Cifrek M., Vidović D. (2023). Borderline and depression: A thin EEG line. Clin. Eeg Neurosci..

[53] Wu W., Zhang Y., Jiang J., Lucas M.V., Fonzo G.A., Rolle C.E., Cooper C., Chin-Fatt C., Krepel N., Cornelssen C.A. (2020). An electroencephalographic signature predicts antidepressant response in major depression. Nat. Biotechnol..

[54] Härpfer K., Spychalski D., Kathmann N., Riesel A. (2021). Diverging patterns of EEG alpha asymmetry in anxious apprehension and anxious arousal. Biol. Psychol..

[55] Kaltsouni E., Schmidt F., Zsido R.G., Eriksson A., Sacher J., Sundström-Poromaa I., Sumner R.L., Comasco E. (2024). Electroencephalography findings in menstrually-related mood disorders: A critical review. Front. Neuroendocrinol..

[56] Surova G., Ulke C., Schmidt F.M., Hensch T., Sander C., Hegerl U. (2021). Fatigue and brain arousal in patients with major depressive disorder. Eur. Arch. Psychiatry Clin. Neurosci..

[57] Ulke C., Tenke C.E., Kayser J., Sander C., Böttger D., Wong L.Y., Alvarenga J.E., Fava M., McGrath P.J., Deldin P.J. (2019). Resting EEG measures of brain arousal in a multisite study of major depression. Clin. EEG Neurosci..

[58] Rodríguez-Ruiz J.G., Galván-Tejada C.E., Zanella-Calzada L.A., Celaya-Padilla J.M., Galván-Tejada J.I., Gamboa-Rosales H., Luna-García H., Magallanes-Quintanar R., Soto-Murillo M.A. (2020). Comparison of night, day and 24 h motor activity data for the classification of depressive episodes. Diagnostics.

[59] Liu S., Liu X., Yan D., Chen S., Liu Y., Hao X., Ou W., Huang Z., Su F., He F. (2022). Alterations in patients with first-episode depression in the eyes-open and eyes-closed conditions: A resting-state EEG study. IEEE Trans. Neural Syst. Rehabil. Eng..

[60] Movahed R.A., Jahromi G.P., Shahyad S., Meftahi G.H. (2021). A major depressive disorder classification framework based on EEG signals using statistical, spectral, wavelet, functional connectivity, and nonlinear analysis. J. Neurosci. Methods.

[61] Rabinovich M.I., Muezzinoglu M. (2010). Nonlinear dynamics of the brain: Emotion and cognition. Physics-Uspekhi.

[62] Nozari E., Bertolero M.A., Stiso J., Caciagli L., Cornblath E.J., He X., Mahadevan A.S., Pappas G.J., Bassett D.S. (2020). Is the brain macroscopically linear? A system identification of resting state dynamics. arXiv.

[63] Ahmadlou M., Adeli H., Adeli A. (2013). Spatiotemporal analysis of relative convergence of EEGs reveals differences between brain dynamics of depressive women and men. Clin. EEG Neurosci..

[64] Tement S., Pahor A., Jaušovec N. (2016). EEG alpha frequency correlates of burnout and depression: The role of gender. Biol. Psychol..

[65] Stacey J.E., Crook-Rumsey M., Sumich A., Howard C.J., Crawford T., Livne K., Lenzoni S., Badham S. (2021). Age differences in resting state EEG and their relation to eye movements and cognitive performance. Neuropsychologia.

[66] Liu Y., Pu C., Xia S., Deng D., Wang X., Li M. (2022). Machine learning approaches for diagnosing depression using EEG: A review. Transl. Neurosci..

[67] Zhu J., Wang Z., Gong T., Zeng S., Li X., Hu B., Li J., Sun S., Zhang L. (2020). An improved classification model for depression detection using EEG and eye tracking data. IEEE Trans. Nanobiosci..

[68] Avots E., Jermakovs K., Bachmann M., Päeske L., Ozcinar C., Anbarjafari G. (2022). Ensemble approach for detection of depression using EEG features. Entropy.

[69] Wu C.T., Huang H.C., Huang S., Chen I.M., Liao S.C., Chen C.K., Lin C., Lee S.H., Chen M.H., Tsai C.F. (2021). Resting-state EEG signal for major depressive disorder detection: A systematic validation on a large and diverse dataset. Biosensors.

[70] Flint C., Cearns M., Opel N., Redlich R., Mehler D.M., Emden D., Winter N.R., Leenings R., Eickhoff S.B., Kircher T. (2021). Systematic misestimation of machine learning performance in neuroimaging studies of depression. Neuropsychopharmacology.

[71] Niles A.N., Dour H.J., Stanton A.L., Roy-Byrne P.P., Stein M.B., Sullivan G., Sherbourne C.D., Rose R.D., Craske M.G. (2015). Anxiety and depressive symptoms and medical illness among adults with anxiety disorders. J. Psychosom. Res..

[72] Bierman E., Comijs H., Jonker C., Beekman A. (2007). Symptoms of anxiety and depression in the course of cognitive decline. Dement. Geriatr. Cogn. Disord..

[73] Judd L.L., Akiskal H.S. (2003). Depressive episodes and symptoms dominate the longitudinal course of bipolar disorder. Curr. Psychiatry Rep..

[74] Bartels S.J., Drake R.E. (1988). Depressive symptoms in schizophrenia: Comprehensive differential diagnosis. Compr. Psychiatry.

[75] Mitchell A.J., Vaze A., Rao S. (2009). Clinical diagnosis of depression in primary care: A meta-analysis. Lancet.

[76] Lynch C.J., Gunning F.M., Liston C. (2020). Causes and consequences of diagnostic heterogeneity in depression: Paths to discovering novel biological depression subtypes. Biol. Psychiatry.

[77] Feczko E., Miranda-Dominguez O., Marr M., Graham A.M., Nigg J.T., Fair D.A. (2019). The heterogeneity problem: Approaches to identify psychiatric subtypes. Trends Cogn. Sci..

[78] Nguyen T.D., Harder A., Xiong Y., Kowalec K., Hägg S., Cai N., Kuja-Halkola R., Dalman C., Sullivan P.F., Lu Y. (2022). Genetic heterogeneity and subtypes of major depression. Mol. Psychiatry.

[79] Drysdale A.T., Grosenick L., Downar J., Dunlop K., Mansouri F., Meng Y., Fetcho R.N., Zebley B., Oathes D.J., Etkin A. (2017). Resting-state connectivity biomarkers define neurophysiological subtypes of depression. Nat. Med..

[80] Ma S., Kang L., Guo X., Liu H., Yao L., Bai H., Chen C., Hu M., Du L., Du H. (2021). Discrepancies between self-rated depression and observed depression severity: The effects of personality and dysfunctional attitudes. Gen. Hosp. Psychiatry.

[81] Acharya U.R., Oh S.L., Hagiwara Y., Tan J.H., Adeli H., Subha D.P. (2018). Automated EEG-based screening of depression using deep convolutional neural network. Comput. Methods Programs Biomed..

[82] Li X., La R., Wang Y., Niu J., Zeng S., Sun S., Zhu J. (2019). EEG-based mild depression recognition using convolutional neural network. Med. Biol. Eng. Comput..

[83] Uyulan C., Ergüzel T.T., Unubol H., Cebi M., Sayar G.H., Nezhad Asad M., Tarhan N. (2021). Major depressive disorder classification based on different convolutional neural network models: Deep learning approach. Clin. EEG Neurosci..

[84] Uyulan C., de la Salle S., Erguzel T.T., Lynn E., Blier P., Knott V., Adamson M.M., Zelka M., Tarhan N. (2022). Depression diagnosis modeling with advanced computational methods: Frequency-domain eMVAR and deep learning. Clin. EEG Neurosci..

[85] Rudin C., Chen C., Chen Z., Huang H., Semenova L., Zhong C. (2022). Interpretable machine learning: Fundamental principles and 10 grand challenges. Stat. Surv..

